# Blood, race and indigenous peoples in twentieth century extreme physiology

**DOI:** 10.1007/s40656-019-0264-z

**Published:** 2019-06-13

**Authors:** Vanessa Heggie

**Affiliations:** University of Birmingham, Birmingham, UK

**Keywords:** Blood, Acclimatisation, Race science, Adaptation, Physiology, Altitude

## Abstract

In the first half of the twentieth century the attention of American and European researchers was drawn to the area of ‘extreme physiology’, partly because of expeditions to the north and south poles, and to high altitude, but also by global conflicts which were fought for the first time with aircraft, and involved conflict in non-temperate zones, deserts, and at the freezing Eastern front. In an attempt to help white Euro-Americans survive in extreme environments, physiologists, anthropologists, and explorers studied indigenous people’s bodies, cultures, and technologies. This paper will sketch an outline of the science of white survival in three ‘extreme’ environments: the Antarctic and Arctic; high-altitude; and the Australian desert, with a particular focus on the ways in which indigenous populations were studied, or in some cases ignored, by Western biomedical scientists—despite their crucial and systematic contributions to the success of experiments and expeditions. Particularly focusing on altitude, and on blood in both its symbolic (hereditary) and literal sense, the article shows how assumptions about race, indigeneity, civilisation, and evolution shaped the ways White Westerners understood their own bodies as well as those of the people they encountered in cold, high and hot places on the earth. Despite new discoveries in physiology and evolutionary science, old racialised assumptions were maintained, especially those that figured the temperate body as civilised and the tropical body as primitive; and in at least one case it will be shown that these racialised assumptions significantly altered, if not retarded, the science of respiratory physiology.

## Introduction

The ways in which certain places ‘make’ certain kinds of human bodies—even certain kinds of human minds—has been an issue of economic and political sensitivity for centuries. Long before Francis Galton coined the phrase ‘nature versus nurture’, doctors, explorers and natural philosophers debated an alternative version of that binary, trying to pick apart the role of environments—‘nature’—in forming human physique, personality, and predisposition to various diseases. So far historians have generally considered these debates in the period of European colonial expansion and consolidation, from around 1600 to the end of the nineteenth century (Anderson 1992; Arnold 1996; Osborne 2014; Seth 2018). These works have shown that there was a clear, but complicated, relationship between medical theories that proposed that sickness and disease was shaped by climate and environment, and natural philosophical theories about the essential differences (if any) between the various bodies encountered during the age of exploration. Put bluntly: did Africans suffer from different diseases to Europeans because they lived in a different climate, or because they were a different kind (species?) of human being? As Suman Seth has recently shown, while such arguments could be part of very localised debates over (medical and scientific) authority, the ‘emergence of modern conceptions of race’ was part of a process that was built on ‘the division of the world into “tropical” and “temperate” disease zones’ as part of a colonial enterprise—not only through the production of something eventually called ‘tropical medicine’ but also through studies of ‘seasoning’, whereby European bodies became accustomed to life in non-temperate zones (Seth 2018, p. 11).

By the eighteenth century this question of ‘seasoning’ was a matter of significant economic significance. As Osborne has argued, ‘acclimatisation’—like seasoning, this means the adaptation of organisms for new environments—was the ‘paradigm colonial science’ (Osborne 2014). While this includes the possibility of growing important cash crops, from sugar to spices, in old or new territories, it also spoke to the challenge of governing new territories. While Seth has nuanced the claim of a sudden shift in racial thinking around 1800, there is nonetheless a significant alteration of theories of acclimatisation through the nineteenth century. A combination of bitter experience and new evolutionary theories combined to create a more pessimistic outlook on the possibility of White survival in non-temperate places. The general sense that there were distinct, different, and fixed biological *races* (as opposed perhaps to more flexible understandings of ethnicities or nationalities), was reinforced by nineteenth century evolutionary theories; these also added the complication that human evolution, the process by which environments exerted their pressures and human bodies became adapted to climates, was now thought to happen over the course of hundreds or thousands of generations, rather than in the lifetime of one person or one family (Harrison 1996; Livingstone 1999; Stepan 1987). When set against anti-colonial uprisings and conflicts, the experience of pandemics such as cholera, and the dramatic mortality and morbidity statistics of military personnel and colonial governors, particularly in the ‘white man’s grave’ of West Africa, it seemed possible that seasoning was a myth, and that the White body was not capable of adapting to non-temperate climates; likewise it seemed possible that the inhabitants of the non-temperate zones were so biologically shaped by their climates that they were doomed to a lower place on the evolutionary hierarchy, for example made ‘lazy’ and ‘uninventive’ by fertile environments and high temperatures (Bolt 1971; Curtin 1989; Kennedy 1990; Stepan 2001).

An important shift happened in these views around the turn of the twentieth century, with the emergence of Tropical Medicine, a quintessentially colonial medical specialism that drew a divide between the ‘tropics’ and ‘normal’ climates, and promised—largely with technological interventions such as vaccines or pesticides—to control the diseases that had ravaged colonial interests (Anderson 1996, 2000; Chakrabarti 2014; Harrison 1999; Worboys 1976). While this phenomenon has attracted plenty of scholarly interest, it has effectively over written the other side of White survival: seasoning, or as it is more commonly referred to in contemporary physiology, acclimatisation. This loss is a mistake, as this article will demonstrate, because the issue of human acclimatisation to new environments remained not only an active research question through the twentieth century, but also one with military, political and economic significance. While colonial motivations still spurred research into White acclimatisation and survival there were also other motivations for studying how White bodies might live and work (and fight) in climates that they had only rarely encountered before: in the first half of the twentieth century expeditions successfully reached all three poles, North, South, and the ‘third pole’ of Everest, and all of these endeavours were surrounded by both directed and coincidental research projects aimed at discovering the right clothes, food and other supportive technologies that would get one nation’s team to the goal before their rivals. At the same time, the Armed Forces of Europe and North America and their allies found themselves fighting in non-temperate climates that clearly demonstrated the weakness of their scientific and technological understanding of survival in heat, cold, and at altitude. World War II in particular involved zones of intense conflict in tropical and desert environments, and the science of cold survival became crucial due to the brutal experiences of the Eastern front and the cold water immersions that attended naval warfare. And, of course, the evolution of ‘war in the air’ was placing human bodies in environments they had never previously encountered; while military research did not always map neatly onto civilian/exploration applications [for example lessons from aviation were misapplied to mountaineering in the early twentieth century (Heggie 2013)] it was still the case that money, attention, and experienced personnel slipped between exploration physiology and aviation physiology from the 1920s onwards. Despite over a century of imperial research into White survival, military medical officers and strategists (and explorers) found themselves with alarmingly scarce and inconclusive evidence about best practice dealing with extreme heat and cold, low oxygen partial pressure, or even fatigue (Adolph 1947). As a consequence money, and perhaps as importantly a huge number of human guinea pigs, was made available to answer basic questions about thermal comfort, sweating rates, hydration, and acclimatisation. War even proved to be a stimulus for work into race and blood: research into blood transfusion and rejection boosted understandings of blood groups and markers, which were subsequently used as biometric tools to understand human history—to trace ancestry and understand how different races and/or ethnic groups evolved (Bangham 2014; Bangham and de Chadarevian 2014; Radin 2017).

Using the specific example of ‘extreme physiology’ (a term referring to the study of the human body at what are believed to be the limits of survival or of acclimatisation) this article argues that race and place, physiology and evolution, remained intertwined through the twentieth century, and that assumptions about whose body ‘belonged’ where had significant impacts on the practices and conclusions of scientific work. It takes altitude physiology as a case study because this was a field in which the study of blood as a literal bodily fluid clearly intersected with the understanding of ‘blood’ as a notion of hereditary, racial identity. Obviously the history of twentieth century race science has been the subject of an enormous amount of scholarly work; of particular relevance here is the work that followed Nancy Stepan’s claims that the ‘old’ ideas of biological race disappeared in the middle of the twentieth century, replaced by a new, more fluid understanding of heredity with a focus on population genetics, statistics, and evolutionary studies of adaptation to environment (Stepan 1987). While undoubtedly the new sciences of molecular biology and (population) genetics influenced understanding of the reality (or otherwise) of race, these forms of identity, privilege, and discrimination were—as Kim TallBear has emphatically shown in the case of the conversion of ‘blood talk’ into ‘gene talk’ in understanding native American tribal identities—a co-construction in the twentieth, as it had been in the nineteenth century: ‘racial science inform[ed] societal thinking about race, [and] popular ideas about heredity and hierarchy… informed the pursuit of scientific knowledge’ (TallBear 2013, p. 34).

This article is an indication that despite the significant scholarship in this field there remains work to be done and co-constructions to uncover. Much attention has been paid to the hereditary and molecular sciences, and far less to the role of alternative biomedical disciplines, particularly physiology. While blood, as a material of heredity, has attracted the scholarship of those interested in surveys of human diversity in the later twentieth century (Bangham 2014; Radin 2017), this tends still to emphasise the role of population genetics. Meanwhile, Warwick Anderson has made the case for anthropology as a co-producer of evolutionary theories in the first half of the twentieth century, but the role of (particularly physical) anthropology in the second half remains largely unexplored (Anderson 2006; although note for example Tilly 2010 on the ‘use’ of anthropology to colonial and imperial interests, at least in Africa). That physiology, even animal physiology, could be used by *anti*-racist scientists, particularly in the context of demolishing claims about environmental determinism, has been demonstrated by Hagen, amongst others (Hagen 2017); but as I have shown (Heggie 2019), and will demonstrate further below, these were minority views through the middle of the twentieth century. This article is a gesture towards the ways in which physiology played a role in reinforcing racial hierarchies and supporting new evolutionary theories; the full dimensions of that relationship remain to be explored, particularly how they functioned in a period which other historians have characterised as after a ‘retreat’ (Barkan 1992) or ‘overturning’ (Farber 2003) of scientific racism in the 1920s and ‘30s—but the sections below outline one element of this story.

## Cold is technology; heat is biology

Travel guides and medical texts of the nineteenth century are replete with advice about adjusting the body and personal habits to deal with non-temperate climates. But despite this interest, despite the centuries of experience of sending military forces across the globe, it is clear that early twentieth century armies and navies felt their understanding of survival science was inadequate for the purposes of modern warfare. Consequently European and American military researchers studied the effects of sudden cold immersion, water and food deprivation, and the combined effects of humidity, heat, and physical labour in the field. Even basic questions remained unanswered, wrote the physiologist EF Adolph in his 1947 book *Physiology of Man in the Desert*: ‘To our knowledge, no systematic presentation of man’s requirements for water has been made heretofore, nor of what happens when they are not met’ (Adolph 1947, p. 17). Immediately this Western research ignored the experience of indigenous desert populations—Adolph goes on to insist that ‘[t]he need for scientific investigation arose [only] when armies began to invade the desert’ (Adolph 1947, p. 17).

Such research into heat survival was sporadic and heterogeneous across the twentieth century, but research into cold was increasingly structured around established research posts and organisations. As with heat, despite more than a century’s experience of exploration in the Arctic, and a half century in the Antarctic, Western explorers and scientists discovered they did not yet have good evidential bases for their choices of clothing, technology, or even food for survival in the far North or extreme South. The formal colonisation of these spaces led to institutes that coordinated and directed such research: for the British the founding of the British Antarctic Survey in 1945 (as the Falklands Islands Dependency Survey, hence FIDS/BAS) was a key moment for Antarctic and cold weather research. Although primarily focused on military intelligence, geopolitical strategy, and geophysical research, FIDS/BAS of necessity also involved research into physiological problems faced by temporary visitors to cold environments, from clothing to infectious diseases to rations. For North America the founding of the Arctic Research Laboratory by the US Navy at Point Barrow in 1947, seems to have been an important starting point for research into acclimatisation, although it mostly focused on fairly basic studies and animal physiology (Hagen 2017; Shelesnyak 1948); more sophisticated, and then controversial (particularly in the case of radiation experiments on indigenous populations) was the US Army’s Arctic Aeronautical Laboratory, which moved from an unlikely start in Texas to Fairbanks in August 1947 (Farish 2013). For Australia the key moment was the forming of Mawson Station—the Australian National Antarctic Research Expeditions (ANARE) base—in 1954 (Kawaja 2013), while for many other nations it was the International Geophysical Year (IGY) of 1957–1958 which acted as the spur to their cold-weather, and specifically Polar, research programmes. While the IGY was focused on climatic, meteorological and geophysical research, it was also an opportunity for team doctors and physiologists to engage in systematic studies of the human body in extreme polar conditions.

Yet despite this interest, funding, and infrastructure, the question of adaptation to cold remained confusing and controversial throughout the twentieth century. What seemed on the surface to be a fairly basic physiological query—do visitors to cold weather climates become acclimatised to low temperatures—proved almost impossible to conclusively answer. As a review (based on ANARE research) outlined in 1964.

Many studies seeking evidence of human acclimatization to cold have been carried out… [the results show that] Following a period of exposure to cold, the subject’s ability to maintain deep body temperature… during acute cold stress has been found to be enhanced, unchanged, and diminished. The metabolic response to cold has been observed to increase, to remain unchanged, and to decrease. Skin temperature during cold stress has been found to be raised, unchanged, and lowered. (Budd 1964, pp. 5–6)

So while a general consensus formed that the peripheries of the body—hands, face and possibly feet—showed some acclimatisation to low temperatures, the question of broader physiological alterations remained uncertain.

Some researchers turned to evolutionary and racial explanations for these findings, which I will discuss below; but others found flaws in the experimental practice in the field. Most obviously, even workers and explorers in the Antarctic did not consistently experience cold stress, after all, the point of the research into buildings and clothing was to create effective micro-climates within the chill of the Arctic or Antarctic. Further, many of the human subjects studied in the polar regions were undertaking hard work during the day, to the point that Antarctic workers were the subjects of a study of *heat* acclimatisation in the late 1960s or early ‘70s: ‘During the hard physical work associated with sledging in the Antarctic, in spite of the low environmental temperatures, it is usual for men to sweat. It seemed possible that a degree of heat acclimatization might be demonstrated as a result of arduous dog-sledge journeys’ (it was) (Wilkins 1971, p. 15). Even where researchers could find subjects repeatedly (if not consistently) exposed to extremely low temperatures over a sustained period of time, it proved extraordinarily difficult to analyse and explain the data that such work generated—the classic example being the doctor Allan Roger’s study of cold adaptation, which involved a 13 year delay between gathering information during the IGY and finally managing to analyse it for publication (Rogers and Sutherland 1971).

Rogers was the team doctor on the Commonwealth Trans-Antarctic Expedition (TAE) of 1955–1958, an attempt to cross the entire continent led by (Sir) Vivian Fuchs from Britain, and the New Zealander (Sir) Edmund Hillary (fresh from his conquest of Everest in 1953). The TAE had volunteered their bodies ‘for science’—as their expedition coincided with the IGY it was folded into the first physiological expedition to Antarctica, INPHEXAN (INternational PHysiological EXpendition to ANtarctica), where British, American and one German researcher studied the scientists and explorers in the Antarctic as part of a large-scale project into acclimatisation and cold stress, with side projects on seal blood, bacteriology and other ‘pet’ projects (Heggie 2019, chapter 3; Tuckey 2013, Chapter 19). Although Rogers’ cold study was an independent piece of work, he collaborated with the physiologists on the expedition, and his was eventually one of the few pieces of work actually published (despite its ‘all star’ team of physiologists INPHEXAN produced remarkably little in terms of publications or conclusions). Rogers had planned to collect records of the clothing worn by expedition members and see if they wore less clothing for equivalent temperatures at the end of the nearly 15 month cross-polar journey, proving that they had become adapted to lower temperatures. In fact the task of converting the mass of data, including weather observations, sickness and exercise records, and multiple daily outfit changes, proved too much for statisticians; it was not until the US Airforce gave Rogers a grant to hire Mrs RJ Sutherland, a mathematics graduate and computing expert, that he found someone capable of wrangling the data into a form that could be calculated. Even then the discovery was that his hypothesis was unproven: despite the extreme circumstances of the TAE there was no evidence of acclimatisation to cold in the White, temperate-zone bodies of the expeditionary team (Rogers and Sutherland 1971).

Despite the difficulties and the ambiguous and variable results, studies into the possibility of short-term acclimatisation did not entirely end in the 1960s—for example the French-led International Biomedical Expedition to the Antarctic (1981) tried forms of artificial pre-acclimatisation using cold baths in late 1979, only to find that the main effect these had was that the explorers ‘got fed up’ (Rivolier 1988, p. 150). But generally speaking researchers in the Arctic and Antarctic began to focus more on other forms of environmental stress: circadian rhythm disturbances, wilderness injuries such as frostbite or snowblindness, or the psychological disturbances of lack of daylight, isolation and ‘cabin fever’. As the consensus grew that acclimatisation to cold was complex, ambiguous, likely non-existent or at best marginal and easily lost, the question remained about how permanent residents of cold environments managed in these spaces. Clearly some populations lived long-term in situations of cold stress to which White bodies could not adapt (either permanent cold, or in the cases often cited of the ‘Australian aboriginal’ or ‘Terra de Fuegians’ of the contrast of very cold overnight temperatures compared with high daytime desert conditions); so were these populations shaped by these environments, showing a hereditary—*racial*—adaptation to non-temperate conditions? (A note on terminology here: I will be using actors’ terms, including ‘Eskimo’, ‘Inuit’ and ‘Circumpolar peoples’, all of which have been challenged and rejected by indigenous groups in the Arctic circle.)

Although nineteenth century racial theorists often represented the peoples of the far North as ‘primitive’ or ‘savage’, they usually dedicated less time to discussing the effects of colder-than-temperate climates on the physical or mental evolution of peoples, compared with the discussions of hot climate populations (more on this below). By the era of intense Arctic exploration, surrounding attempts at the North Pole around the early twentieth century, the focus was more on the anthropological study of ‘Eskimos’ than the physiological study of their bodies—considering the technological and social solutions (ice houses, migratory living patterns) that indigenous peoples developed to cope with cold conditions. Two of the most influential researchers into circumpolar life, the Canadian Vilhjalmur Stefansson (1879–1962) and Norwegian Kåre Rodahl (1917–2008) played down the idea that ‘Eskimos’ were racially adapted for the cold (Stefansson 1912, 1946; Rodahl 1964; Farish 2013). Stefansson in particular championed the theory that the traditional high-meat diet of indigenous people led to a raised metabolic rate, in other words, the body was capable of producing more heat through muscular activity. This was not a special adaptation by a particular ethnic group: on a traditional ‘western’ diet Eskimo metabolism fell to ‘normal’ European levels, and vice versa, Europeans found their metabolism began to rise on an ‘Eskimo diet’. Rodahl, a generation later, drew similar conclusions. Essentially the early twentieth century studies of ‘Eskimo’ physiology and adaptation suggested that the practice of ‘going native’—as discussed by Cavell—of adapting indigenous practices and technologies, would render non-indigenous bodies as resistant to the cold as those of the permanent residents of the far North (Cavell 2009).

Even when detailed genetic studies became possible, their focus was often on the origins of Circumpolar populations and their evolutionary history, rather than trying to identify specific, inherited, physiological adaptations to cold weather. Perhaps the largest example is the International Biological Programme (1964–1974), a 10-year project inspired by the IGY that intended to provide a similar survey of the organic (rather than inorganic) features of the Earth. One strand of this research was the ‘Human Adaptability Section’, under which was subsumed all research into the physiology of human bodies, as well as their genetic and cultural heritage. Overwhelmingly, the research under this programme considered long-term adaptation to environments, that is, hereditary physiological adaptations, ideally ones that could be used as genetic markers to trace the evolutionary and migratory history of the human race. It was grounded in a strong assumption of environmental determinism and Darwinian Natural Selection—that climates would leave indelible marks on the bodies of human beings, and that ethnic groups would be somewhat, if not ‘perfectly’, adapted to their ecological niches (Aronova et al. 2010). It was also, as Joanna Radin has shown, a place where, in TallBear’s terminology ‘blood talk became gene talk’, where there was a slippage between discussions of ‘untouched’ and ‘primitive’ peoples—the assumption being that some living populations, exclusively non-White, were literally closer to primitive shared human ancestors, so that molecular biology could become a form of evolutionary archaeology (TallBear 2013; Radin 2017). And yet even in this fairly racialised atmosphere the assumption that human bodies evolved for their environments proved difficult to sustain with regard to the populations in the Arctic.

Of course, Arctic populations suffered from the same confounding factors as did temporary visitors to the two poles: they created microclimates, with shelter and clothing, that mitigated against the cold of their environments. Indeed, as multiple studies through to the 1970s showed, ‘Eskimo’ clothing (generally read to mean based on animal furs) often proved superior in its thermal capacity than the wool, cotton, and later artificial fabrics of Western scientists and explorers (Heggie 2019, chapter 3). If White bodies were failing to acclimatise properly because their cold exposure was limited by survival technology, the same could be argued for the indigenous populations of the Arctic. Hopes that evolutionary studies would still reveal adaptations evolved by ‘ancient’ Arctic populations who did not have such advanced technology, were eventually dashed: the summation of the IBP’s research in this area (*The Human Biology of Circumpolar Populations*, published in 1980) showed no evidence had been found to disprove the much older theories of Steffanson and Rodahl that any ‘adaptations’ of Circumpolar peoples (e.g. raised thyroid activity or metabolic rate) were entirely explained by cultural factors, such as diet and working patterns (Milan 1980). Any genetic ‘differences’ that identified such groups—for example blood group distribution, general physique, height—were the result of random genetic flow and other features of non-directed population genetics, and were not a response to environmental pressures. The indigenous peoples of the Arctic Circle had responded to their environment with technological and cultural innovations, not physiological responses.

This conclusion, that human beings deal with cold via technological innovation, did not come as a surprise in 1980. In trying to explain the ambiguous results of acclimatisation studies at mid-century many scientists had already turned to theories of human evolution:

Man, as an animal whose original habitat was the tropical forest, might already have reached the limits of his physiological adaptation to cold in moving to ‘temperate’ climates, so that ‘normal’ man is in fact almost maximally cold-acclimatized. (Budd 1964, p. 6)

This refigured the temperate zone as the ‘limit’ for human acclimatisation (and notice how much work ‘normal’ is doing here; bodies acclimatised to anything other than the temperate zone are ‘not normal’). To go beyond this required technological innovation, or at least basic survival technology, and this was the case for all bodies, regardless of their racial or ethnic identity. This conclusion about the cold, however, left the opposite condition—heat stress—open to question. Could ‘normal’ man adapt to heat?

Heat and cold were often surprisingly interlinked—as seen above in the use of Polar explorers to study fatigue and heat during hard work—and this sometimes drew the populations of hot and cold climates together. In *The Human Biology of Circumpolar Populations* the Japanese physiologist Sinji Itoh suggests that the nomadic Lapp’s ability to sleep in very cold temperatures may be due to a ‘response to a moderate cold stress [that] resembled that found in the Australian Aborigines’ (Milan 1980, p. 287). This cold resistance had been noted in Australian populations earlier in the century, and had been a contentious issue for researchers. Anthropological and physiological study of Aboriginal populations had evolved out of anthropometrical studies at the turn of the twentieth century (Anderson 2006). Usually ranked ‘lowest’ in racial hierarchies, the Australian Aboriginal was widely expected, by colonial administrators and scientists alike, to become extinct when faced with the competitive presence of White settlers. In the face of high racial tensions, the ‘White Australia’ movement, and policies such as the removal of children of mixed-race parentage, a strand of more sympathetic physical anthropology and physiology emerged (Bashford 2000). While some of this remained deeply paternalistic, concerned about the impact of ‘Western’ civilisation on ‘untouched’ people (particularly in relation to alcohol and Western food habits), increasingly physiologists began to consider how it was that these ‘backwards’ people survived in the uncompromising environments across the Australian continent (and to connect ‘heat’ and ‘cold’ adaptation more directly, see Powell 1980 for an account of a debate between the arctic expert Stefansson, who believed the ‘arid centre’ could be colonised, and the more pessimistic race scientist Griffith Taylor).

Studies into the acclimatisation of White bodies to heat started in earnest during the Second World War; as with cold acclimatisation they immediately ran into the dual challenges of trying to measure a multifactorial environmental stress and a holistic bodily response. While some standard, and immediate, adjustments could be measured in visitors to hot climates—such as sweating rates—it was unclear how far these adaptations could be pushed, how long and sustained a heat stress exposure needed to be in order to develop long-term physiological changes, or whether studies in hot chambers doing ‘artificial acclimatisation’ mirrored the experience of living in the tropics (increasingly researchers thought that fatigue, or at least exercise, was an important co-component in the process of acclimatisation, so resting tests in climate chambers were declared insufficient, or poor models for the ‘real world’ (Fox et al. 1963).

As with cold climates, researchers turned to indigenous populations as a comparator: if it was difficult to mimic adaptation in climate chambers, if the results of studying temporary or short-term residents were ambiguous, perhaps answers could be found in the bodies of people who had lived in hot climates for generations. Of course this would only be a partial answer to the question of *White* survival; even if physiological adaptations could be found in the bodies of indigenous peoples, the question then remained open whether these were adaptations that any body could achieve simply by long term exposure to a hot climate, or whether they were hereditary adaptations, exclusive to some racial groups and developed over centuries, not weeks and months. Many researchers concluded that ‘the indigenes’ were not ‘fully’ acclimatised to heat, perhaps partly because acclimatisation requires exertion, or deliberate exposure to heat stress and ‘man does not normally choose to exert or expose himself sufficiently to develop his capacity of heat acclimatisation to the full’ (Fox et al. 1974). By the early 1970s it remained unclear whether short-term and long-term (e.g. physiological and evolutionary) responses to heat stress were essentially distinct or part of a spectrum, and one can read the frustration of researchers as they admitted that conclusively identifying ‘true ethnic differences’ of response would be ‘as difficult and baffling as trying to discover how it is that two individuals of the same race, age, occupation and physical build, living in the same climate, can differ so widely in their responses to heat’ (Fox et al. 1974; p. 76).

Despite the ambiguities of heat acclimatisation research, it clearly remained easier to believe that adaptations to heat were biological, as opposed to the technological adaptations to cold. While researchers acknowledged the diversity of racial and ethnic groups living in warmer-than-temperate climates, there are specific instances of tension that demonstrate a clear preference for biology as an explanandum for survival advantages in hot environments. Specifically, the Australian outback was a space where blood, climate, physiology, race, and nationhood came together; As Warwick Anderson has shown, by the late 1920s the working hypothesis for Aboriginal survival seems to be one based on environmental determinism. Indigenous Australians were assumed to have a long term, hereditary adaptation to heat survival (a question mark hovered over their ability to sleep in the extremely cold night climate of the Australian deserts). This was not a unanimous assumption, however, and by mid-century there were physiologists actively resisting this narrative, for example the animal and human physiologist Walter Victor MacFarlane (1913–1982). In a summary of his findings in an undated manuscript from the late 1970s or early 1980s he describes the ‘tropical origins of man’, tracing the Aboriginal line back to the Asian tropics. Consequently, ‘[Aboriginals] have retained the high sweat rates and water dependence of tropical peoples’, which means that *just like circumpolar people*, they have adapted to the climate through behavioural and cultural, not physiological processes.^1^ They arrange societies around water sources, drink large quantities in one sitting, and engage in physically active processes in the cooler parts of the day.

This was, essentially, a positive message for those concerned with White survival. As Macfarlane argued later the ‘population explosion’ of the mid-twentieth century would put pressure on the ‘uninhabited’ regions of Africa, Indo-China, Australia and South America, and there was reason to be positive that these spaces could be healthily inhabited (MacFarlane 1965, p. 55). The ‘normal’ man was biologically adapted to hot humid climates (as were the Australian aborigines), and temperate-climate dwellers could therefore habituate to higher temperatures (‘[e]xposure to hot environments, especially from infancy, seems to be the best means of adjustment to tropical living’ (MacFarlane 1965, p. 63)). For hot *dry* climates the challenges could be tackled by paying attention to the cultural and technological solutions of indigenous peoples, particularly the ability of ‘desert aboriginal nomads’ to ‘water load’—that is, drink vast quantities of water, and habituating their systems to reduced urination (MacFarlane 1965, p. 58). (This is, in fact, advice given to contemporary travellers, with the rough shorthand that it is better to carry water inside you, where it can help you, than outside you, where it drains your energy).

And yet, looking across the research into heat and cold, it is clear that it remained easier—almost instinctive—to believe that cold weather adaptations were cultural and technological, while hot weather adaptations were biological, possibly racial. At the same time as Bantu blood and Singaporean sweat and Aboriginal water cultures were being scrutinised by Western physiologists, a debate was raging about the origins of the human race in all its forms. The middle of the century is when the ‘Out of Africa’ theories of human origin and dispersal began to seriously challenge the ‘Out of Asia’ hypothesis. The ways in which the physiological study of human acclimatisation fed into shifts in evolutionary theory is a topic for another work, but here it is clear that a physiological model that sought out biological adaptations to heat (whether dry or tropical), and assumed that the temperate man was already maximally acclimatised to cold and could only ‘go colder’ with technological support, fitted neatly with overarching beliefs about the deep history of the human race(s). It also, of course, engaged with racist beliefs about the hierarchy of races and ethnicities. While Western commentators usually celebrated the ability to innovate to survive, to use technology to conquer environmental threats, there was also anxiety about the apparent superiority of other peoples in extreme climates. Particularly within the context of rugged, exploratory, or military masculinity, the fact that non-White peoples tolerated higher or lower temperatures, showed less fatigue or hunger or stress, was a constant theme in writing about the hot, cold, and high parts of the earth. One way to solve this anxiety about the superiority of other races was to label it as *biological*; the ‘noble savage’ remained unchangeably shaped by his environment, while temperate man had evolved into his civilised status. We have already seen this reflected in the IBP’s work on ‘Human Adaptability’ where it is the ‘untouched’ populations of the tropical zone that are often read as literally being closer to, and therefore more representative of, primitive human populations and shared ancient ancestors (Aronova et al. 2010). This tension, between superior physical performance (or at least a survival advantage) and technological innovation is perhaps most clearly seen when it clashed in one single ‘extreme’ environment: high altitude.

## High

The fact that visitors to mountainous regions experience strange symptoms—headaches, fatigue and shortness of breath being the most common listed—has been noted by many writers, across the world, and hundreds of years ago when Paul Bert the so-called ‘father of respiratory physiology’ sat down to write his magnum opus about altitude physiology, he started with a long ‘Historique’ section which included experiences of ‘headache mountains’ and breathlessness as far back as the sixteenth century (Bert 1878; West 1998). Bert himself was more interested in exploring the phenomenon of ‘mountain sickness’ in a controlled laboratory setting which, as I have demonstrated, was part of an emerging trend of reductive, laboratory physiology in the nineteenth century (Heggie 2013). Bert’s extensive laboratory testing, published as a major survey of the topic *La Pression Barométrique* in 1878, forcefully argues that the issue was not altitude per se, but the reduced oxygen partial pressure present at altitude; consequently supplemental oxygen could be used to prevent or ‘cure’ mountain sickness. Despite his own focus on the laboratory, some of his work was based on older field research, particularly that by his medical colleague and funder, Denis Jourdanet (1815–1892), who had spent around 18 years in Mexico, and had developed an interest in the diseases of high altitude. Jourdanet’s many works in this area included ‘*De l’anémie des latitudes et de l’anémie en general dans ses rapports avec la pression de l’atmosphère*’, which he published in 1863 and which used field observations and laboratory tests to suggest that the problem of altitude was lower arterial oxygen concentration (Kellogg 1978; West and Richalet 2013).

Jourdanet had noticed the similarity between mountain sickness and various anaemic diseases—coining the term ‘anémie barométrique’ for the syndrome, which included dizziness and nausea along with the traditional symptoms of breathlessness and fatigue (West and Richalet 2013). But, puzzlingly, while patients with sea-level anaemia typically had pale, watery blood, those at altitude often had dark coloured and thick, sometimes even viscous, blood. The cause of this, and the fact that it was a reaction to altitude was confirmed in 1890 when (with Bert’s encouragement) the French researcher François-Gilbert Viault (1849–1918) measured the concentration of red blood cells in both visitors to, and indigenous residents of, the Andes [and in native wildlife (Windsor and Rodway 2007)]. Both temporary and permanent residents at altitude had blood cell concentrations (‘haematocrit’ measurement) above the ‘normal’ standards found in sea-level populations. This was a particularly exciting finding as it indicated a single, and universal, response to the environmental challenge posed by increased altitude. Both (long term) evolution and (short term) physiological acclimatisation produced the same result in the human body—an increased concentration of red blood cells in the blood. This explained Jourdanet’s findings too, ‘barometric anaemia’ was caused not by an inherent problem in the body, but rather by the environment itself—and the body’s own homoeostatic mechanisms had a defence against this environmental challenge: it significantly increased the red blood cell concentration which would in turn help to transport more of the (scarcer) oxygen from the lungs to the rest of the body.

While Bert’s theory about reduced oxygen partial pressure did not achieve total consensus in Western physiology until the early twentieth century [for example, the influential Italian researcher Angelo Mosso (1846–1910) maintained that carbon dioxide was the crucial gas, evidenced by high profile deaths on Alpine climbs that were not prevented by inhaling oxygen (Felsch 2007)], but it was consistently the dominant theory. It was accepted by the key British researchers, including Joseph Barcroft and J.S.Haldane, both of whom engaged in expeditions to mid-altitude to test their theories about respiration and circulation. While British climbers started testing oxygen systems in the Himalaya in the 1920s, it was South and Central America that most often drew scientists from Europe and North America who were interested in studying and alleviating the phenomenon of altitude sickness, or *soroche*. In 1921 Joseph Barcroft led an expedition to Cerro de Pasco in Peru, where a mining company kept workers at a permanent residence some 4300 m above sea level, then believed to be the highest permanent settlement in the world (West 1998). Much of the work Barcroft and his team did in Peru in 1921 was turned into material for his 1925 book *The Respiratory Function of the Blood*, particularly *Part I: Lessons from High Altitudes* in which he makes a rather startling claim about high-altitude populations: ‘[a]ll dwellers at high altitudes are persons of impaired physical and mental powers’ (Barcroft 1925, p. 176; West 2016).

Such a claim fell on politically fertile soil, as Marcus Cueto has emphasised, coinciding with a rise in ‘Peruvian nationalistic ethos’ (Cueto 1989, p. 646) and firing one researcher in particular to a defence of the Andean peoples. Carlos Monge Medrano (1884–1970), claimed that this quote was a driving motivation for his extensive research programme into the physiology of mid and high altitude residents: he led or organised eight expeditions to various Andean regions in the late 1920s and early 1930s, and became the director (1934–1956) of the *Instituto de Biología y Pathología Andina* (Cueto 1989, p. 643; West 2016). Monge Medrano’s concerted research programme aimed to visit the same communities as Western researchers had used to better understand the ‘truth’ of what he came to call the ‘Andean man’, and indeed to defend their physical robustness and superiority against claims of imperfection and inadequacy, and particularly to argue that using sea-level measures of ‘normal’ performance or physiological markers was inappropriate (Cueto 1989).

As the introduction to this article suggested there was, by the first half of the twentieth century, a well-established tradition in Western biomedicine of environmentally determinist theories about the relationship between people and place; often this shored up fundamentally racist assumptions about the superiority of one group over another. While much historiography has focused on nineteenth century physiological understandings of environmentally determined races, degeneration, and ‘primitiveness’, often as a part of a justification of colonial or even genocidal practices, Anderson (1996, 2000, 2006) amongst others, has demonstrated the continuation of these themes well into the twentieth century. After all, Barcroft’s Cambridge colleague, the physiologist and geneticist J.B.S. Haldane—son of J.S. Haldane with whom Barcroft had a long-running dispute over respiration physiology—suggested as late as the 1930s that immigration between Africa and Europe should be discouraged because ‘Negros’ were ill-adapted to temperate climates, while Europeans’ physiology could not cope with ‘the tropics’ (Stepan 1987, p. 154).

And yet, when read in context, Barcroft’s claim about the ‘impairment’ of altitude populations turns out to be less deterministic than the isolated quote might suggest. His argument is rather that the essence of acclimatisation to any new environment is the ‘redistribution of disadvantages’, that is, in a homoeostatic system any adaptation to a new environment will inevitably have knock-on consequences, or be some form of physiological compromise. While this doesn’t address Monge Medrano’s central complaint that there was no reason to take the sea-level body as the ‘normal’ body, and regard others as variations (just as there was no reason to presume that the temperate zone body was ‘normal’ and the tropical zone a variant or a primitive form), it is not intended as a critique of populations at altitude. Barcroft was not comparing indigenous altitude populations to sea-level populations and finding the former lacking, but rather comparing any one individual’s performance at altitude compared to their performance at sea-level; indeed, in this scenario bodies adapted to the low oxygen conditions at altitude might perform *better* at sea-level than indigenous sea-level populations—a principle later adapted into forms of sports training.

Given this reinterpretation of Barcroft, it is slightly ironic that as part of his critique Monge Medrano went on to write a deeply environmentally deterministic account of the history of South and Central America, which attempted to explain the history of the continent and its peoples in large part as a function of adaptation to environmental pressures—what he referred to as ‘climatic aggression’. Translated and published as *Acclimatization in the Andes: Historical Confirmations of ‘Climatic Aggression’ in the Development of Andean Man* in 1948, Monge Medrano’s work argues that there is a specific racial type living at high-altitude—the ‘Andean Man’ of the title. This ‘race’ of human is different enough from the sea-level type that it justified measurement ‘with a scale distinct from that applied to the men of the lower valleys and plains’, e.g. assumptions about ‘normal’ haematocrit or arterial oxygen measurement should be reconsidered (Monge 1948, p. xii). Monge M. makes the case for Andean Man as a kind of superman, a natural athlete and epitome of health (and we should acknowledge that there is little information in the book about Andean *woman* as a category). But his book was also—as is so often the case for racial science—a deliberate political intervention. Monge M. mobilised his expertise in physiology to push more broadly into debates about race, identity and nationality; too broad to detail here, in summary he uses his studies to criticise policies on public health, the movement of labour, economic development, and general social trends in Peru and across the continent. Later, as Cueto has shown, this argument for the ‘Andean man’ fell out of favour, and the work was absorbed into more generic ‘high altitude studies’ (Cueto 1989).

But there is evidence in his writing that Monge M. intended his ‘Andean Man’ to be a broader category than merely the residents of the Andes; there are suggestions of a universal racial type for mid and high altitude populations. The logic of environmental determinism suggests that the same ‘climatic aggression’ in different places should lead to the same (racial) adaptations. So, for example, Monge M. asserts that the Tibetans are of a similar physiology—strong, hearty, resistant to altitude sickness, and insensible to physical pain. This is despite the fact that by 1948 virtually no physiological or biomedical work had been done on the populations of the Himalaya. Although the superior climbing skills of the people who became known as Sherpa were recognised by European climbers in the late 1910s, they were not enrolled into the physiological studies that backed up and accompanied the frequent European attempts on Everest, Kanchenjunga and Nanga Parbat. Instead this work was done on indigenous peoples in Central and South America, while Europeans and White Americans tested their own bodies in the Andes, Alps, Himalaya and Rockies (Tracy 2012). What these studies confirmed time and time again was Viault’s original assertion—that all bodies adapt in at least one universal way to the ‘aggression’ of climate, by increased haematocrit.

Debates over the mechanism and degree of polycythaemia (increased red blood cell counts) continued well into the middle of the twentieth century. There are two ways, after all, to increase the concentration of red blood cells—either increase the number of red blood cells by upregulating production (and possibly downregulating destruction) but also by reducing the liquid content (plasma) of the blood. Since mountaineers are often dehydrated—not just because of the effort of climbing and heavy breathing, but also the logistical difficulty of transporting clean water and the dryness of many mountain atmospheres—this remained a complicating factor in the assessment of haematocrit changes at altitude. Although a possible hormonal mechanism to upregulate red blood cell production was posited in the first years of the twentieth century, the hormone responsible, Erythropoietin, was not specifically identified until the 1970s [and only synthesised in the 1980s, at which point it became a powerful agent for sports doping (Gleaves 2015)]. Nonetheless, despite some uncertainties it seemed logical, in 1948, to assume that indigenous peoples at mid-altitude in the Himalaya would adapt in a similar way to those in the Andes; the more pressing question seemed to be whether such adaptation could only be achieved over generations, or whether sea-level bodies, who already displayed acclimatisation using the same mechanism, could achieve similar performances to Sherpa guides by long-term residence at altitude.

This faith in environmental determinism—an assumption that ‘climatic aggression’ would create similar bodies everywhere—was both incorrect, and somewhat lazy. Although Himalayan peoples had not been recruited into Western scientific projects to anywhere near the same extent as Andean populations, one British doctor *had* examined their blood as early as 1924, and his finding was both startling, and completely ignored. T. Howard Somervell was the team doctor to the British Everest Expedition of 1924—the expedition during which George Mallory and Andrew ‘Sandy’ Irvine disappeared during a summit attempt. The British had been experimenting with supplemental oxygen as a means to deal with altitude sickness since the first (reconnaissance) Everest expedition in 1921, and Somervell attempted to conduct some physiological research on the mountain, in order to better understand the responses of the human body to the reduced oxygen partial pressure of altitude. In a short article in the *Journal of Physiology* he reported on measurements of oxygen and carbon dioxide in samples taken from the Everest team, but the most original finding of his study is mentioned only in the very last line of the article:

The colour indices of two Tibetans, taken at 16,500 feet, at which height most of their lives had been spent, were 92 and 82; remarkably low figures for men who can race up steep slopes about twice as fast as we could with our colour indices of 120. (Somervell 1925, p. 285)

The ‘colour indices’ mentioned here are a way of measuring haematocrits—roughly speaking the darker the blood, the higher the colour index, and the higher the concentration of red blood cells/level of polycythaemia. Somervell had found that polycythaemia is *not* a universal human response to altitude.

There are two explanations why Somervell’s findings had little impact on altitude physiology despite the fact that the subsequent decades were period of extreme interest in field physiology and the study of indigenous peoples (Heggie 2019). First is the pragmatic—Somervell’s article had a serious methodological flaw when it came to his work on oxygen and carbon dioxide. He had used football (soccer) ball bladders to collect his air samples from the British climbers, and these proved to be porous to carbon dioxide, throwing his figures and calculations into doubt. Maybe in that circumstance his closing lines about Sherpa blood were also dismissed as a clear error of experimental practice. But it is also the case that there was extraordinarily little effort made to duplicate his studies. This was partly to do with the comparative difficulty of conducting studies in Nepal or Tibet—due to both geographical and political challenges—which made these less favourable regions compared to the infrastructure and local support for respiratory science in South America (at least by the 1930s). It was also caused by a pervasive attitude that the Sherpa people would resist attempts at study and scrutiny, and could not be trusted to take part in scientific work (Ortner 1999). As late as 1958 one of the world’s leading altitude physiologists, the Dr Lewis Griffith Cresswell Evans Pugh (who had been responsible for much of the research that made the 1953 attempt on Everest successful), suggested that even a cardiological survey of Sherpa villages would be difficult because the local people would not be as co-operative as ‘more educated’ Western people (or, presumably, Andean man).^2^ In fact Pugh himself did involve a few Sherpa people in his studies, around Everest in the early 1950s and as part of the so-called ‘Silver Hut’ physiological expedition of 1960–1961 (Heggie 2019)—and his work confirmed Somervell’s findings, and marked the start of a changed theory of adaptation to altitude. Yet he, and other researchers, continued to complain that they were not good experimental subjects—for example, apparently only one Sherpa learned to use a bicycle well enough to provide useful data, as an ergometer was the main technology used to measure work output during physiology experiments (Pugh 1962, p. 624).

The sparse samples of Sherpa blood shifted understandings of respiratory physiology; by the second half of the twentieth century physiologists had begun to speculate that polycythaemia is actually a *disadvantage* in high-altitude situations. Part of the ‘redistribution of disadvantage’ Barcroft had warned about, ‘thicker’ blood circulates less easily in small vessels, and can increase the risk of frostbite and anoxia in the peripheries of the body. Indeed by the 1970s mountaineers were experimenting with ‘blood-thinning’ technologies as forms of performance enhancement [although it turned out that merely ensuring that climbers were not dehydrated was as effective—possibly more so—than complicated and risky infusions of plasma to ‘dilute’ the blood (Zink et al. 1982)]. The relative under study of the adaptational features of high-altitude populations in the Himalaya continued through the middle of the twentieth century; it was only in 2017 that British scientists published the first analysis of Sherpa physiology that explained how they could perform so well with so few red blood cells (Horscroft et al. 2017). There is obviously a counterfactual history to be explored here: how would blood and respiratory physiology have changed if Sherpa had been the models for ‘normal’ adaptation, not Andean residents and White bodies? But as significant is the demonstration of the relationship, even in late twentieth century science, between race, place and (literally) blood—that the assumption of universal and environmentally determined physiological features was not something physiologists were motivated to challenge.

## Conclusion

The American publishers of Monge Medrano’s book, the Johns Hopkins Press, commented on the fact that his extreme ‘environmental determinism’ may seem ‘unfashionable’ to Western scientists. Yet despite this disclaimer it is clear that assumptions about the effects of ‘climatic aggression’ on human bodies as being inevitable and universal did not seem to provoke theoretical refutation, nor encourage further confirmatory study of indigenous populations (as has been illustrated here specifically by the absence of Sherpa from physiological study in the first half of the twentieth century, despite hints that they may provide brand new insights into human biology). It is also clear that these assumptions about homogeneity, as well as of difference, were used to reinforce conclusions developed by much older forms of racial science: of the sturdy native who does not feel pain, of the superiority of the cold climate peoples over those of the tropics.

The climates, and the bodies they apparently shaped, considered here are at the extremes of human survival. As such they could be dismissed as exceptional and uninformative about broader attitudes to ‘normal’ human evolution and adaptation. And yet, contemporaries did not struggle to see the potential value of such studies to the bodies of those born in temperate climates: it is notable that, while much of this research was conducted by civilian scientists, it was frequently dependent on military funding. While some of this is the canny exploitation of available grants by scientists themselves, it is also obvious that the US military in particular saw the value of physiological studies not only of White acclimatisation, but also of non-white adaptation to extreme environments. Macfarlane’s work on the ‘Water Economy of Desert Aboriginals and New Guinea Melanesians’ in the 1960s was funded by a grant from the US Army Medical Research and Development unit; Allan Rogers was only able to process his data on cold adaptation with a grant from the US Air Force; and as Jordan Bimm and others have shown, physiologists working on similar topics managed to fund their work by making appeals to the needs of the Space Race (Bimm and Killan 2017). The interrelation of White acclimatisation, and non-white adaptation therefore remained an issue of colonisation and conquest well into the late twentieth century; however ‘exotic’ the bodies or climates studied, race and place remained intertwined in everyday assumptions about the way human bodies respond, in the short and long-term, to their environments.

## Footnotes

1 University of Adelaide Rare Books & Special Collections [Hence: Adelaide]. *W.V. Macfarlane* *Papers 1947*–*1985 (MS0006),* Undated Manuscript ‘Water, Salt and Food for Tropical medicine’.

2 Pugh Papers, Mandeville Special Collections—University of California, San Diego. Letter G Pugh to PO Williams [MRC] 25 Nov 1958.
